# The Pattern of Social Parasitism in *Maculinea teleius* Butterfly Is Driven by the Size and Spatial Distribution of the Host Ant Nests

**DOI:** 10.3390/insects14020180

**Published:** 2023-02-12

**Authors:** Magdalena Witek, Valentina La Morgia, Luca Pietro Casacci, Francesca Barbero

**Affiliations:** 1 Museum and Institute of Zoology, Polish Academy of Science, Wilcza 64, 00-679 Warszawa, Poland; 2 Institute for Environmental Protection and Research (ISPRA), Via Ca’ Fornacetta 9, 40064 Ozzano Emilia, Italy; 3 Department of Life Sciences and Systems Biology, University of Turin, Via Accademia Albertina 13, 10123 Turin, Italy

**Keywords:** ant colony size, host specificity, join count statistics, myrmecophily, multiparasitism, *Maculinea*, *Microdon myrmicae*, spatial association

## Abstract

**Simple Summary:**

*Maculinea* butterflies are endangered social parasites of *Myrmica* ants. In late summer, caterpillars abandon their foodplants and wait for a *Myrmica* worker to retrieve and carry them into the nest. Here the caterpillars spend 11 months consuming the ant brood or being fed by workers; then they pupate early in summer, and in one month the adults emerge. Our study aimed to assess the spatial relationship between nests parasitized by *Maculinea teleius* and those unparasitized and the factors influencing the parasite presence inside host nests. We searched for *Ma. teleius* caterpillars in ant nests in autumn, during the initial larval development, and in the following late spring. Unsurprisingly, we found a substantial decrease in the proportion of parasitized nests from autumn to late spring. The biggest *Myrmica* nests adopted a higher number of parasites, but mid-size nests provided the best trade-off between competition and resource availability, leading to high parasite survival observed in spring. The spatial distribution of parasitized nests in autumn was uniform, while the colonies in which *Ma. teleius* survived until pupation were grouped. Overall, our results suggest that host colonies’ features and spatial relationships should be considered when trying to preserve these rare butterflies.

**Abstract:**

The parasitic relationship between *Maculinea* butterflies and *Myrmica* ants has been extensively studied but little information is available on the spatial occurrence of *Maculinea* larvae. We searched for the presence of *Maculinea teleius* in 211 ant nests at two sites in two crucial phases of its life cycle, i.e., in autumn, during the initial larval development, and in the following late spring, before pupation. We assessed variations in the proportion of infested nests and factors correlated with spatial distributions of parasites in *Myrmica* colonies. The parasitism rate in autumn was very high (∼50% of infestation rate) but decreased in the following spring. The most important factor explaining parasite occurrence in both seasons was the nest size. Further factors, such as the presence of other parasites, the *Myrmica* species or the site, concurred to explain the differential survival of *Ma. teleius* until the final development. Irrespective of the host nest distribution, the parasite distribution changed from even in autumn to clumped in late spring. Our work showed that the survival of *Ma. teleius* is correlated with colony features but also with the nest spatial distribution, which therefore should be taken into consideration in conservation strategies aiming at preserving these endangered species.

## 1. Introduction

Many social insects, such as ants, live in colonies representing a forcefully protective environment as well as an abundant source of food. These traits make ant nests suitable to host many arthropods, either occasional visitors or steady guests fully dependent on the colony resources [1,2,3,4]. Obligate social parasites belong to the latter group, and, according to a broad definition, they are intruding arthropods that exploit any resources of an ant colony for some phases of their life cycle [5].

*Myrmica* Latreille, 1804 (Hymenoptera, Formicidae) ants are hosts to many parasitic ant species, primarily members of the same genus [6,7,8,9,10,11,12,13] but are also known for hosting immature instars of many other insects, such as *Maculinea* butterflies [14,15,16]. The survival of *Maculinea* Van Eecke, 1915 (Lepidoptera, Lycaenidae) larvae depends on the presence of their host ant nests but also on a specific food plant [17,18]. In Poland, females of *Maculinea teleius* Bergsträsser, 1779, the species surveyed in this work, lay their eggs in July-August, specifically on *Sanguisorba officinalis* Linné, 1753 (Rosales, Rosaceae). After feeding for three weeks on the foodplant, *Maculinea* larvae fall to the ground, where they are adopted by *Myrmica* workers thanks to the implementation of deceiving strategies based on multimodal signals [19], both chemical [20] and acoustical [21]. Once inside the host colony, larvae of *Ma. teleius* display a predatory strategy feeding on the ant brood, usually choosing the largest *Myrmica* larvae [22].

All *Myrmica* species that forage in the area beneath *Maculinea* food plants have the same probability of adopting butterfly larvae. Still, the survival probability of these larvae varies, depending on the adopting *Myrmica* species [23]. Many recent studies on host ant specificity have shown that *Ma. teleius* is among the most generalist species of its genus [15,24,25,26,27] and can survive inside nests of all available *Myrmica* species. Nevertheless, *Myrmica scabrinodis* Nylander, 1846 is the most common host of *Ma. teleius* populations in Europe [15].

Ant colony size is another critical factor playing a crucial role in the social parasite’s survival. By studying another *Maculinea* predatory species, *Ma. arion* Linné, 1758, Thomas & Wardlaw [22] estimated that *Myrmica sabuleti* colonies must contain a minimum number of 354 workers to rear one butterfly larva. Therefore, only a few *Myrmica* nests can support these parasites in nature. Inside host nests, *Ma. teleius* and *Ma. arion* larvae are subject to scramble competition because they compete for a finite resource which is equally accessible. Thus, the parasite survival rate drops along with the increase of caterpillar density in the same colony, often leading to only one or two *Maculinea* predatory larvae surviving until pupation [22].

Although the host and parasite relationship between *Maculinea* butterflies and *Myrmica* ants has been extensively studied, little information is available on the spatial pattern of *Maculinea* larvae occurrences with respect to their host ant nest distribution. In addition, the spatial pattern observed in the autumnal, first phase of adoption and in the post-hibernation phase have never been compared yet. In *Ma. teleius,* adult females evenly lay eggs on *Sanguisorba officinalis* and avoid food plants already carrying conspecific eggs [28]. Since *S. officinalis* is usually very abundant at sites with *Ma. teleius* [29], at least at the beginning of their larval development (autumn) parasitic larvae inside host colonies are also supposed to show an even distribution. In contrast, the parasite distribution found in late spring is linked to the survival of the parasites, which varies according to colony size, resource supply status and species identity of *Myrmica* nests [30].

In our study, we investigated factors affecting the rate of infestation of *Myrmica* nests and the micro-spatial distribution of the infested host colonies. In detail, the main aims of our study were to assess: (i) the spatial distribution of all *Myrmica* colonies, as well as of those infected by *Ma. teleius*, (ii) the infestation rate of *Myrmica* host nests, (iii) the influence of host species, nest size and presence of other competitors, e.g., larvae of other *Maculinea* species or *Microdon myrmicae* Schönrogge et al., 2002 (Diptera, Syrphidae) syrphid fly, on the occurrence of *Ma. teleius* larvae inside *Myrmica* colonies. We chose to focus our study on the occurrence of *Ma. teleius* because this parasite uses various *Myrmica* species as hosts, showing a high infestation rate that provides sufficient data to feed the statistical models. Moreover, the broad spatial distribution of *S. officinalis* allows for assessing potential variation in the parasite survival patterns from an initial even distribution due to its food plant occurrences.

We assessed spatial patterns of larval distribution within nests at the beginning of autumn, the parasite’s initial colonisation phase, and late spring when fully-grown *Maculinea* larvae are about to pupate. We expect infestation rates to be higher in autumn than in the following spring because of the high mortality rate faced by overwintering butterfly stages. Our hypothesis is that the colony size is a key factor affecting the presence of *Ma. teleius* larvae, with larger *Myrmica* colonies containing more parasitic larvae. Therefore, we envisage differences in *Ma. teleius* spatial distribution between the two phases of its life cycle, with an even distribution in autumn and a clumped pattern in spring, driven by intra-nest competition.

## 2. Materials and Methods

### 2.1. Study Areas

Studies were conducted at two sites: (1) Kosyń, in eastern Poland (51°23′ N/23°34′ E; 161 m a.s.l.) and (2) Kraków, in southern Poland (50°01′ N/19°53′ E; 220 m). Both sites are wet meadows dominated by *Molinia* Schrank, 1789 (Poales, Poaceae) spp. and are characterised by different communities of social parasites of *Myrmica* ants: (1) *Maculinea teleius*, *Maculinea nausithous* Bergstrasser, 1779 and *Microdon myrmicae*, in Kosyń; (2) *Ma. teleius*, *Ma. nausithous*, *Maculinea alcon* Denis & Schiffermüller, 1776 and *Mi. myrmicae*, in Kraków. *Sanguisorba officinalis*, the food plant of *Ma. teleius* (and *Ma. nausithous*), occurs at both study sites, and its density is 16 and 6 plants per m^2^, respectively, in Kraków and Kosyń. *Gentiana pneumonanthe* Linné, 1753 (Gentianales, Gentianaceae), the larval food plant of *Ma. alcon*, is present only in Kraków.

In both populations, adults of *Ma. teleius* are on the wing between the end of June and the end of August [31].

### 2.2. Field Survey

**Figure 1:**
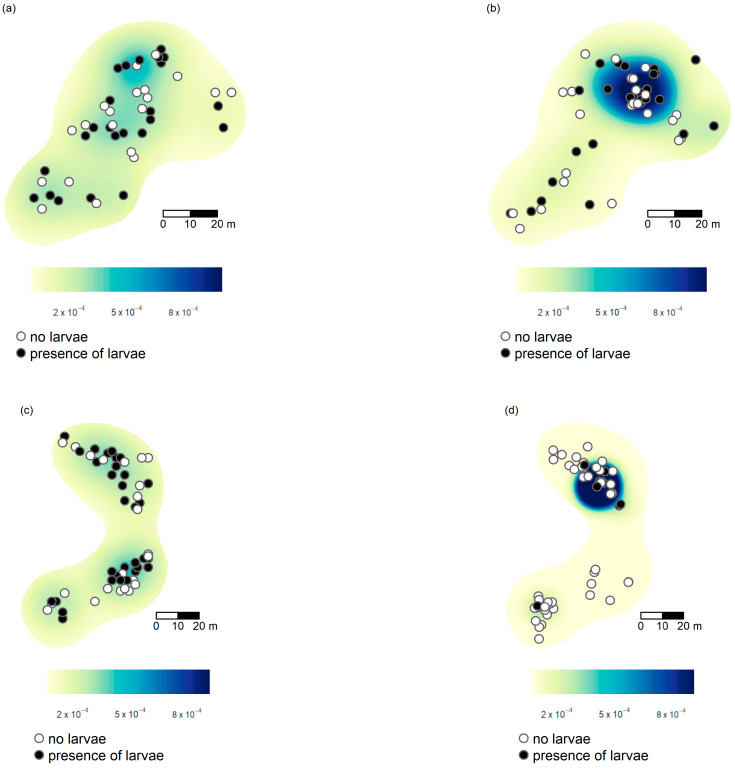
Distribution of infested (black dots) and non-infested (white dots) *Myrmica* nests in Kraków during (**a**) autumn and (**b**) late spring and in Kosyń during (**c**) autumn and (**d**) late spring. The Kernel-smoothed probability density of larvae is shown in the background (colour shades).

Data were collected at the beginning of October (hereafter “autumn”) and again in the middle of June (“late spring”), i.e., in the initial part and at the end of *Ma. teleius* development inside ant host colonies, respectively. Each area was an irregularly shaped grassland of 0.42 ha in Kosyń and 0.43 ha in Kraków (Figure 1). Within the grassland, *S. officinalis* was present in sub-areas with homogenous coverage. We surveyed for the presence of *Myrmica* ants by conducting a scrutiny search, sensu [32], along 2 m-width transects scattered only on the area covered by the food plant *Sanguisorba officinalis*. Therefore, all examined nests could be potentially infected by *Maculinea teleius*.

In our studied sites, *Myrmica* colonies build their nests in tufts of grass, which usually have different chimney sizes. Firstly, after finding the nest, data on ant nest size were collected following the method described by Nash et al. [33]. In brief, the number of *Myrmica* workers emerging when the nest was first opened (after splitting the grass tuft centre for the first time) were counted, allowing the classification of colonies into small (less than 20 workers), medium (20–100 workers), and large (>100 workers). Later, the nest was open, and all brood chambers were inspected for the presence of social parasite larvae. If necessary, we partially excavated the nest to reach the brood chamber. Thus, we counted *Ma. teleius* preimaginal instars, used as the dependent variable, and larvae or pupae of other social parasites (i.e., *Maculinea* spp. and *Microdon myrmicae*) as explanatory variables. From each ant colony, 10–20 workers were collected and preserved in 70% ethanol. The analytic key of Czechowski et al. [34] was used for ant species, while we used the key by Śliwinska et al. [35] for *Maculinea* larvae identification. The position of each *Myrmica* nest was determined by Garmin GPSMAP 60CSx and further recorded on a map. Information on the nest number and the composition of *Myrmica* ant communities, along with the infestation rates observed at the two study areas, is shown in Table 1.

### 2.3. Statistical Analyses

Chi-square tests were used to compare the degree of nest infestation between autumn and the following spring, for all studied populations. To analyse nests’ spatial distributions, we first considered the location of each ant nest within a 4 × 4 grid superimposed to the map of the study area grids, irrespectively of the ant species and of the presence/absence of social parasites’ larvae. We determined whether the pattern was consistent with Complete Spatial Randomness (CSR) using Monte Carlo quadrat tests and by calculating the Variance Mean Ratio (VMR) at different spatial scales, multiples of a 4 × 4 m sampling grid superimposed to the map of the study area for the analyses. We preferred quadrat count-based statistics to nearest-neighbour analyses since quadrat counts were more robust to errors in geolocation. Two-sided Monte Carlo tests were performed by generating 999 expected counts, according to a CSR hypothesis and comparing the corresponding Pearson chi-square statistic with the one for the observed point pattern. Secondly, we analysed nests’ association patterns via Monte Carlo tests based on random labelling and join count statistics [36,37]. Join count statistics (J) test whether or not the occurrence of categorical attributes at spatially adjacent sampling locations can be accounted for by randomness alone. To establish adjacency, the spatial neighbourhood of each nest was defined as the subset of the other nests falling within predefined, increasing distances, matching the scales of previous quadrat counts. For all categorical attributes (see further below), we directly performed the calculation of join count statistics, since a simple analysis of departure from CSR would be affected by the spatial distribution of nests. This approach was adopted to (1) detect patterns of association between *My. scabrinodis* nests (dominant *Myrmica* species) and those of other, less abundant *Myrmica* ant species, by calculating the join count statistics for pairs of adjacent *My. scabrinodis* nests (J_ss_), pairs of nests of other *Myrmica* species (J_oo_) and pairs with one nest of *My. scabrinodis* and one nest of other *Myrmica* species (J_so_); (2) test the significance of aggregation of big/medium sized nests, by join count statistics focused on the J_bb_ statistics (pairs of big and medium nests as opposed to small nests); (3) test the spatial association of ant nests with and without social parasites (I - infested nests; E - empty nests, see also further below for details). The observed values of join count statistics were compared with those obtained from random re-labelling (999 replicates) of the ant nests, i.e., nest locations were fixed but labels indicating ant species/nest-size/parasitic infestation were redistributed [38]. To assess possibly positive spatial associations between nests of the same type (i.e., the same ant species/the same size or the same infestation state), we hypothesised that join count statistics should be higher than expected (“greater” hypothesis). The Monte Carlo *p*-value was thus estimated as: (random values equal to or greater than the observed one + 1)/(random values + 1). As concerns the other join count statistics (e.g., association of nests of different ant species, association of nests with different parasites), we were also interested in assessing possibly negative spatial association (or repulsion). In these cases, we tested the hypothesis that joint count statistics were lower than expected (“less” hypothesis) and the Monte Carlo *p*-value was estimated as: (random values equal to or less than the observed one + 1)/(random values + 1). The spatial distribution of nests belonging to different size groups was considered, since nest size was of interest for subsequent modelling (see further below). For infested nests, Monte Carlo analyses were performed (a) by grouping all infested nests regardless of the social parasite, (b) separately for *Maculinea teleius* and (c) to test for positive or negative association between *Ma. teleius* and larvae of other social parasites. All spatial statistics were calculated separately for each study site and sampling period. We also visually assessed distributions by plotting kernel smoothed probability density maps. These maps were obtained by R [39] adehabitatHR package [40], by estimating the smoothing parameter with the default ad hoc method [41].

The next step was to use several candidate mixed regression models to explain the occurrence of *Ma. teleius* larvae inside *Myrmica* colonies. We took “autumn” data collected in the initial part of the parasites’ larval development to reflect the ability of *Myrmica* colonies to adopt parasitic larvae, whereas data collected in late spring of the following year provided an indication of long-term conditions for larval survival within the nest. Thus, considering the same initial set of explanatory variables, we separately fitted models for autumn and late spring data to investigate possibly different seasonal processes. In particular, we related the presence of *Ma. teleius* larvae to the following fixed effects (1) *Myrmica* host ant species—a categorical variable with two levels: *My. scabrinodis* or other, less abundant, *Myrmica* species; (2) nest size—a categorical variable with three levels: large, medium, and small nests; (3) study site; (4) presence/absence of other social parasite larvae; (5) interaction between the nest size and presence/absence of other parasites. We verified the lack of relevant correlations between explanatory variables, and we then hypothesised various candidate models including different subsets of explanatory variables. We selected variables and interaction terms on a biological basis rather than evaluating all possible models in an automated selection framework, because the latter can result in selecting a “spurious” best model, and we then compared the candidate models in terms of AIC values [42], finally taking into account the regression coefficients obtained by averaging models with ΔAIC < 2. For model checking, given the potential problems arising from spatial autocorrelation of data, we fitted variograms to the residuals of our models, to check whether spatial autocorrelation was likely to impact the analyses [43], or whether to include an appropriate spatial correlation structure [44]. Statistical analyses were performed on R 4.2.2 [39].

## 3. Results

### 3.1. Infestation Rates

In Kraków, *Maculinea teleius* was the most abundant social parasite, both considering the number of infested nests as well as the number of larvae found inside *Myrmica* nests (Table 1). In Kosyń, *Ma. teleius* infested the highest number of *Myrmica* nests in both seasons compared to other social parasites but, in autumn, the highest number of larvae found inside the host nests belonged to *Mi. myrmicae* (Table 1). Only a small number of nests infested by *Ma. nausithous* and/or *Ma. alcon* were found (Table 1). In Kraków, 51% (*n* = 23) of *Myrmica* colonies were infested by larvae of *Ma. teleius* in autumn, and 43% (*n* = 21) in the late spring. For Kosyń the percentage of colonies infested by *Ma. teleius* was 40% (*n* = 23) and 15% (*n* = 9) in autumn and spring, respectively. The proportion of infested nests with first stages of larval development was significantly different than the proportion of nests parasitised by late instars found in the next spring in Kosyń (χ^2^_1_ = 4.53, *p* = 0.03), whereas no difference was observed at the Kraków site (χ^2^_1_ = 0.090, *p* = 0.765).

Irrespectively of the species, the estimated density of *Myrmica* nests was 0.2 nests/m^2^ in Kraków and 0.3 nests/m^2^ in Kosyń. A few *Myrmica* species were present in Kraków and Kosyń, but *My. scabrinodis* was the most abundant at both sites. In Kraków, the latter ant species was exploited by *Ma. teleius*, *Ma. alcon* and *Mi. myrmicae* and in both sampling periods, more than 60% of *My. scabrinodis* nests were infested (*n* = 20 in autumn, *n* = 22 in late spring). The second most abundant species was *Myrmica ruginodis* Nylander, 1846 (Table 1), whose nests (70% in autumn, *n* = 7, and 53% in late spring, *n* = 8) were infested only by *Ma. teleius*. In Kosyń, *My. scabrinodis* nests were infested by *Ma. teleius* and *Mi. myrmicae* with a significantly higher proportion of colonies (χ^2^_1_ = 8.81, *p* = 0.003) infested in autumn (71.1%, *n* = 32) than in spring (17.1%, *n* = 7). A similar pattern was observed for nests of *Myrmica rubra* Linné, 1758, which were infested by larvae of *Ma. teleius*, *Ma. nausithous* and *Mi. myrmicae* and whose infestation rate was higher in autumn (90%, *n* = 9) than in the following spring (50%, *n* = 5) (χ^2^_1_ = 0.23, *p* = 0.026).

### 3.2. Spatial Patterns

The VMRs calculated for each study site and season were always larger than 1, suggesting that the spatial distribution of *Myrmica* nests was clumped. Significant departures from CSR increased with quadrat size (e.g., in Kosyń in late spring, for quadrat width d = 12 m, VMR = 6.73, *p* = 0.002, for quadrat width d = 32, VMR = 8.69, *p* = 0.006). Because of the dominance of *My. scabrinodis* nests at all sites and periods, we performed the association analysis of *My. scabrinodis* with other *Myrmica* ants at both sites. In late spring, we observed a significant positive association of *My. scabrinodis* nests (e.g., at spatial distance d = 12 m, J_ss Kraków_ = 260, *p* = 0.020; J_ss Kosyń_ = 488, *p* = 0.009), and a significantly negative association of *My. scabrinodis* nests with colonies of other *Myrmica* species (d = 12 m, J_so Kraków_ = 74, *p* = 0.016; J_so Kosyń_ = 156, *p* = 0.008). These association patterns were detected at all scales in Kraków, and at small and intermediate scales (d < 20 m) in Kosyń.

**Figure 2:**
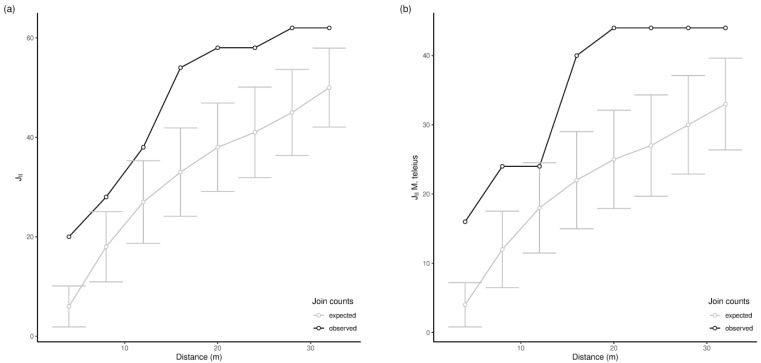
Observed and expected join count values (sum across nests) at different spatial distances (with standard deviation, SD, for expected values) in Kosyń in spring. The plots show the significance of the aggregation of infected nests by considering all parasites (J_II_, panel (**a**)) or only *Ma. teleius* (J_II_ *Ma. teleius*, panel (**b**)). For positive spatial association, observed values must be significantly larger than expected.

Grouping all infested nests, no significant associations of infested or non-infested (hereafter called parasite-free nests) nests were detected in autumn for both Kraków and Kosyń sites (Figure S1a,b). In late spring, positive associations were detected at almost all scales for Kosyń (e.g., d = 16 m, J_II_ = 54, *p* = 0.036; Figure 2a and Figure S1c,d).

**Figure 3:**
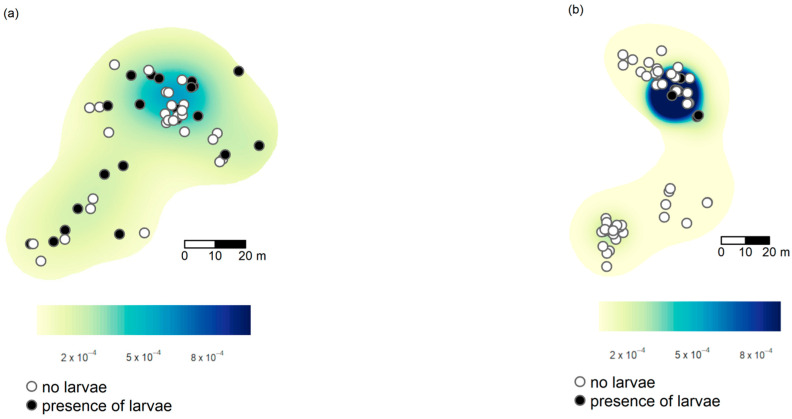
Kernel-smoothed density of *Myrmica* nests infested by *Ma. teleius* larvae, with superimposed locations of *Ma. teleius* larvae, in Kraków (**a**), or Kosyń (**b**), at the end of larval development.

A similar pattern emerged when we considered only the locations of *Ma. teleius*: a significant positive association of *Ma. teleius* larvae was detected at almost all scales in Kosyń (e.g., d = 20 m, J_II *Ma. teleius*_ = 44, *p* = 0.025; Figure 2b, Figure 3b and Figure S2). In Kraków, we also detected an increase in the values of the J_II *Ma.teleius*_ statistics in June (Figure 3a) with respect to October (Figure S2a,b), albeit the differences from expected values were not statistically significant. No patterns were detected when we considered only infested nests and we tested for segregation of *Ma. teleius* against all other larvae (Figure S3).

**Figure 4:**
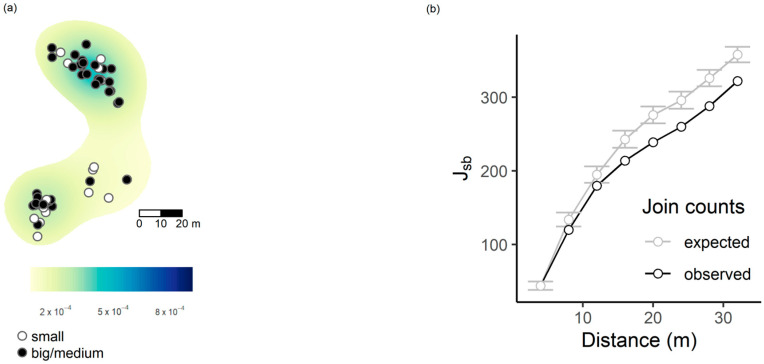
Kernel-smoothed density of large/medium *Myrmica* colonies at the end of larval development, in Kosyń (**a**). Panel (**b**) shows the significance of the segregation (join count statistics, J_sb_) between big-medium nests (grouped together) and small nests. In case of significant segregation, observed values must be smaller than expected.

As concerns join count statistics for ant-colony size, the main result was that, in late spring, in Kosyń we observed significant segregation of big/medium nests (grouped together) with respect to the small ones, especially at intermediate and large scales (e.g., d = 20 m, J_sb_ = 44, *p* = 0.025; Figure 4).

### 3.3. Factors Correlated with the Presence of Ma. teleius and Mi. myrmicae Larvae Inside Myrmica Nests

**Figure 5:**
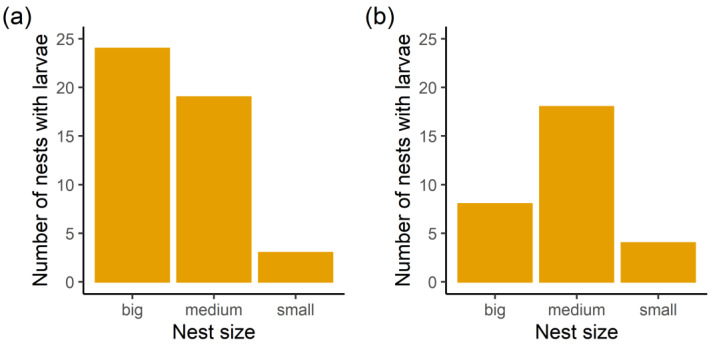
Occurrence of *Ma. teleius* larvae in nests of different size in (**a**) autumn, and in (**b**) spring.

For both seasons and parasite species we selected a base model including all explanatory variables as fixed effects (*Myrmica* species, study site, presence of other parasite larvae, colony size, interaction between colony size and the presence of other parasites, as well as interaction between ant species and the presence of other parasites). We then fitted candidate models including subsets of the explanatory variables, as shown in Table 2. According to information criteria, the most appropriate models (∆AIC < 2) differed among study sites and parasite species. For *Ma. teleius*, colony size was the only variable included in the model generating the lowest AIC in autumn and the estimated regression coefficient of big nests with respect to the small ones was statistically significant (β = 1.731 ± 0.719 SE, *p* = 0.016; Figure 5a). Equivalent models (in terms of AIC) also included site and ant species as explanatory variables (Table 2), but according to model averaging, colony size remained the most important variable (average β_Nest Size: big vs. small_ = 1.721 ± 0.723 SE, *p* = 0.019; average β_Nest Size: medium vs. small_ = 1.01 ± 0.715 SE, *p* = 0.163). At the end of larval development, variables involved in explaining the presence of *Ma. teleius* larvae included colony size, study site, ant species, presence of other parasites and its interaction with the ant species. According to the model with the lowest AIC, significant regression coefficients were detected for the nest size, especially for medium-sized nests (β = 1.428 ± 0.677 SE, *p* = 0.035; Figure 5b), compared to the small ones. When we compared big and small nests, the regression coefficient was β = 1.237 ± 0.845 SE (*p* = 0.143). The presence of *Ma. teleius* differed (β = −1.695 ± 0.562 SE, *p* = 0.003) between the Kosyń and the Kraków site. Accordingly, the colony size and study site were identified as the most important variables by model averaging (average βN_est Size: big vs. small_ = 1.266 ± 0.865 SE, *p* = 0.148; average β_Nest Size: medium vs._ small = 1.416 ± 0.696 SE, *p* = 0.044; average β_Site: Kosyń vs. Kraków_ = −1.613 ± 0.561 SE, *p* = 0.004).

## 4. Discussion

Results presented in this paper indicate that several factors can affect the presence of *Ma. teleius* inside *Myrmica* nests but the most important is the colony size, which is pivotal both during the first phase of nest colonisation, in autumn, and for the parasite survival after the overwintering period, in late spring.

In detail, big colonies are infested more frequently by *Ma. teleius* in autumn and medium colonies in the spring. Indeed, big *Myrmica* colonies may adopt more parasite larvae since they include larger numbers of foraging workers (e.g., [45]), thus increasing the probability of finding *Maculinea* larvae within this kind of nests. It is also possible that bigger nests have higher within-colony genetic variation, perhaps linked to the presence of many queens, and are consequently more prone to social parasitism, thus adopting more larvae [45,46]. The finding that, at the end of their development, *Ma. teleius* larvae are found more often inside medium-sized nests suggests that the parasite survival is, on the one hand, dependent on food resources, still abundant in medium colonies [47] but, on the other side, can be affected by larval scramble competition [22]. The very high infestation rate of big colonies in autumn could lead to extremely high competition among *Ma. teleius* larvae and many (if not all) of them would not survive until the end of their development [22]. Therefore, medium-sized colonies can provide the optimal balance between the availability of resources and the level of scramble competition for the butterfly parasite to achieve the best survival.

In addition to the size, other colony features are correlated to the parasite occurrence, but their contribution is greater in explaining the larval survival in late spring than their initial infestation in autumn. The lack of a significant influence of *Myrmica* species on the presence of *Ma. teleius* larvae at the beginning of the butterfly cycle is consistent with previous studies showing that *Maculinea* caterpillars have the same probability of being adopted by any *Myrmica* species that forage in the surroundings of the food plants [23]. In contrast, we found that the species of *Myrmica* can partially explain *Ma. teleius* occurrence in late spring (Table 2). Still, this is not the most important variable explaining the parasite’s survival and it proves to be crucial when we consider its interaction with the presence of other parasites. This finding is not surprising as *Ma. teleius* is a rather generalist species, able to exploit several *Myrmica* species as hosts [15,24,26,27] while *Mi. myrmicae* and *Ma. alcon* are specialised to exploit only few or one species of *Myrmica* ants locally [15,23,48], primarily *Myrmica scabrinodis* in these two Polish populations. However, these two variables, i.e., *Myrmica* species and the other parasite occurrences, differ between the two sites, contributing to clarify why the “site” is another crucial variable explaining the survival of *Ma. teleius* in late spring. In Kraków three *Myrmica* species, i.e., *My. scabrinodis*, *My. ruginodis* and *My. rubra,* are present and used as hosts by *Maculinea* butterflies. *My. rubra* is very rare (only 5% in autumn and 2% in spring, among all *Myrmica* nests), but *My. scabrinodis* and *My. ruginodis* are abundant enough to compare their infestation rates between the two sampling events. Our results show that a similar proportion of *My. scabrinodis* and *My. ruginodis* nests is infested in autumn and in the end of the parasite development, thereby suggesting that *Ma. teleius* survival is high in both these ant species. This finding could indicate that in Kraków *Ma. teleius* population is truly generalist or that environmental conditions are particularly suitable for *Myrmica* colonies that are more prone to rear the parasite (see further below). In Kosyń, in contrast, the most abundant *Myrmica* species are *My. scabrinodis* and *My. rubra* with the former showing a substantial drop in the rate of parasitism by *Ma. teleius* from 42% in autumn to 7% in late spring. The high survival in the nest of *My. rubra* and the elevated mortality in the nests of *My. scabrinodis* in Kosyń explain the reason why “*Myrmica* species” is listed as a factor in the best models. Such a high survival rate of both *Ma. teleius* and *Ma. nausithous* larvae inside the nests of *My. rubra* can be explained by the highest similarities of chemical profiles between social parasites and this host ant species [49]. Our previous studies performed on the same populations showed that *Ma. teleius* cuticular hydrocarbon profile was 50% similar to that of *My. rubra* and only 38% similar to *My. scabrinodis*, which could suggest higher host specificity of *Ma. teleius* larvae toward *My. rubra* ants.

More in general, the occurrence of *Ma. teleius* in the host nest at the beginning and at the end of the butterfly development differ between the two sites, irrespective of the *Myrmica* species considered. Overall, the pressure of *Ma. teleius* we estimated in our work is very high since about half of the investigated ant colonies are infested in autumn (51% in Kraków and 43% in Kosyń). This scenario greatly changes at the end of the parasite development in Kosyń, where only a small proportion (15%) of host nests is still infested. Surprisingly, in Kraków, the proportion of nests with *Ma. teleius* does not differ between autumn and the following late spring (40%). While the proportion of nests parasitised by *Maculinea teleius* late instars in Kosyń is consistent with data gathered in other European populations [50], the parasitism rate (40%) of the Kraków population is particularly high also compared to previous observations performed in the same site (in 2003 and 2004, 11% and 12% of nests were infested by late *Ma. teleius* instars [27]). This result can be due to particularly benevolent conditions leading to well-fed colonies, which can support a higher number of parasites [22] or natural fluctuations in population size, frequently observed in *Maculinea* butterflies [51]. In addition, a study carried on in the same area showed that the probability of occurrence of *Maculinea* larvae and pupae in *Myrmica* nests was significantly higher in temporarily inundated meadows [52] than in control meadows. Unfortunately, we did not measure soil humidity systematically but, when we performed our field survey, meadows in Kraków were inundated. Even though how the presence of water can affect the ant colony performance is not straightforward, we tentatively concur with Kajzer-Bonk et al. [52] in pointing out that soil humidity may be one of the predictors for the presence of *Ma. teleius* in a mosaic landscape [53], see also 32 for the effect of soil moisture and temperature on ant niche selection.

Conversely, the spatial analysis is consistent at the two sites. If we consider all the *Myrmica* species found in a site, their nest distribution both in Kraków and Kosyń is clumped, even at small spatial scales. The same pattern is found when we compare the nest distribution of the most abundant species, *My. scabrinodis*, with other *Myrmica* species. *My. scabrinodis* nests have clumped distributions and are separated in space from nests belonging to *My. rubra*, *My. ruginodis* or *Myrmica gallienii* Bondroit, 1920. This kind of nest distribution suggests a polydomous structure for *My. scabrinodis* populations [54] but can also reflect distinct microclimatic niche preferences of each *Myrmica* species (e.g., [30,55]). Despite the fact that *Myrmica* colonies are clumped, in autumn, the distribution of *Ma. teleius* infested nests is even and does not show any spatial aggregation, suggesting that the infestation probability [23] is equal for all *Myrmica* nests and did not depend on their location. This result is in line with the expectation that early *Ma. teleius* distribution is based on the female oviposition pattern [28]. The most interesting finding is that in the course of its development we observe a variation in the spatial occurrences of the parasite, suggesting that the survival of *Ma. teleius* is also influenced by the position of the parasitised nest with respect to other *Myrmica* colonies. Although this result is significantly higher in Kosyń, at both sites we estimate an increase in the positive association of infested nests, leading to a change from an even (in autumn) to a clumped distribution of parasitised nests in late spring. This spatial heterogeneity, with patches where social parasite occurrence is higher than in others, is also correlated to the finding that big and medium-sized nests had clumped distributions. In other words, nests are grouped in habitat patches where conditions are apparently suitable to allow *Myrmica* colonies to grow to larger sizes, thereby generating more resources and better conditions for parasitic larval development. We should also mention that *Myrmica* colonies can constitutively differ in their susceptibility to infestation and nests more prone to social parasites may be grouped together as they can represent polydomous structures that promote social parasitism [1]. Overall, our results concur to support the general idea proposed by Hölldobler and Wilson [1] stating that hotspots of social parasites do not arise by chance and are promoted by several, peculiar ecological factors.

## 5. Conclusions

Our work indicates that the survival rate of obligate social parasites such as *Ma. teleius* depends on several ecological factors, among which the host ant colony size proved to be one of the most important. Of course, the colony size itself is affected by several factors such as the particular microclimatic conditions where the nest is found. These small-scale characteristics could eventually also explain the spatial distribution of these “optimal” (from the parasite’s point of view) nests [55]. Therefore, while the parasite occurrence in autumn is mainly driven by the female egg-laying behaviour and uniform host plant spreading, the survival of *Ma. teleius* late instars is correlated with many other variables related to the colony or population structure of the *Myrmica* ants [5]. Finally, the differences observed between the two Polish sites reveal that even though general patterns and paradigms of social parasitism hold across populations, local parasite adaptations to their ant hosts occur and temporal variation in environmental conditions (e.g., soil humidity) could also deeply influence the survival of these rare and endangered parasite species. Hence, conservation strategies implemented to preserve populations of *Ma. teleius* should also consider diverse factors affecting both the colony features but also to the nest spatial distribution.

## Figures and Tables

**Table 1:** Information on *Myrmica* host colonies and their social parasites in two sampled populations. Symbols: T—*Maculinea teleius*, N—*Maculinea nausithous*, A—*Maculinea alcon*, M—*Microdon myrmicae*. Some nests with double infestation were also observed.

Site	Ant Species	No. and (%) of Nests		No. of Infested Nests and (Number of Larvae)	
		Autumn	Late Spring	Autumn	Late Spring
Kosyń	*My. scabrinodis*	45 (79%)	41 (68%)	19_T_ (48), 13_M_ (61)	4_T_ (6), 3_M_ (4)
	*My. rubra*	10 (18%)	10 (15%)	3_T_ (9), 3_N_ (6), 3_M_ (15)	3_T_ (62), 2_N_ (36)
	*My. gallienii*	2 (3%)	9 (17%)	1_T_ (3)	2_T_ (3), 1_N_ (1)
Kraków	*My. scabrinodis*	33 (73%)	33 (67%)	15_T_ (49), 2_A_ (7), 3_M_ (5)	12_T_ (24), 1_A_ (6), 9_M_ (22)
	*My. ruginodis*	10 (22%)	15 (31%)	7_T_ (17)	8_T_ (15)
	*My. rubra*	2 (5%)	1 (2%)	1_T_ (3), 1_N_ (1)	1_T_ (1)

**Table 2:** AIC values for models with different fixed structures fitted for the two study seasons. The base models relate the presence/absence of *Ma. teleius* larvae (dependent variable) to all explanatory variables included as fixed effects. Models selected according to information criteria (∆AIC < 2) are in bold.

	*Ma. teleius* Presence	
Model Structure	Autumn	Late Spring
Ant species × Other parasites + Site + Other parasites × Nest size	146.1	**109.7**
Ant species × Other parasites + Site + Nest size	145.5	**108.0**
Ant species + Site + Nest size × Other parasites	144.2	110.1
Ant species + Other parasites + Nest size + Site	143.5	**108.9**
Ant species × Other parasites + Nest size × Other parasites	145.3	117.5
Ant species + Nest size × Other parasites	143.3	116.6
Nest size + Site + Ant species	142.1	115.8
Nest size + Site	**140.2**	116.0
Nest size + Ant species	**141.0**	125.7
Nest size + Site + Other parasites	141.6	110.9
Ant species × Other parasites	146.6	118.2
Nest size × Other parasites	141.4	119.1
Nest size	**139.0**	126.0
Ant species	144.1	128.4
Site	143.2	121.7
Other parasites	143.8	122.0

## Data Availability

Data are available upon request.

## Supplementary Materials

The following supporting information can be downloaded at: https://www.mdpi.com/article/10.3390/insects14020180/s1, Figure S1: Observed and expected values of the J_II_ statistics based on empty and infested nests, regardless of parasite species; Figure S2: Observed and expected values of join count statistics calculated for nests infested by *Ma. teleius*, relative to empty nests; Figure S3: Observed and expected values of join count statistics calculated for nests infested by *Ma. teleius* and/or other parasites.
