# Supplementary material for: The Pattern of Social Parasitism in Maculinea teleius Butterfly Is Driven by the Size and Spatial Distribution of the Host Ant Nests
Source: Insects. 2023 Feb 12;14(2):180. doi: 10.3390/insects14020180 (PMC9961401; doi:10.3390/insects14020180)

Figure S1. Observed and expected values (sum across nests with SD) of the J_{II} statistics (based on empty and infested nests, regardless of parasite species) calculated for autumn at Kraków (a) and Kosyń (b), and for spring (c) at Kraków and (d) at Kosyń.

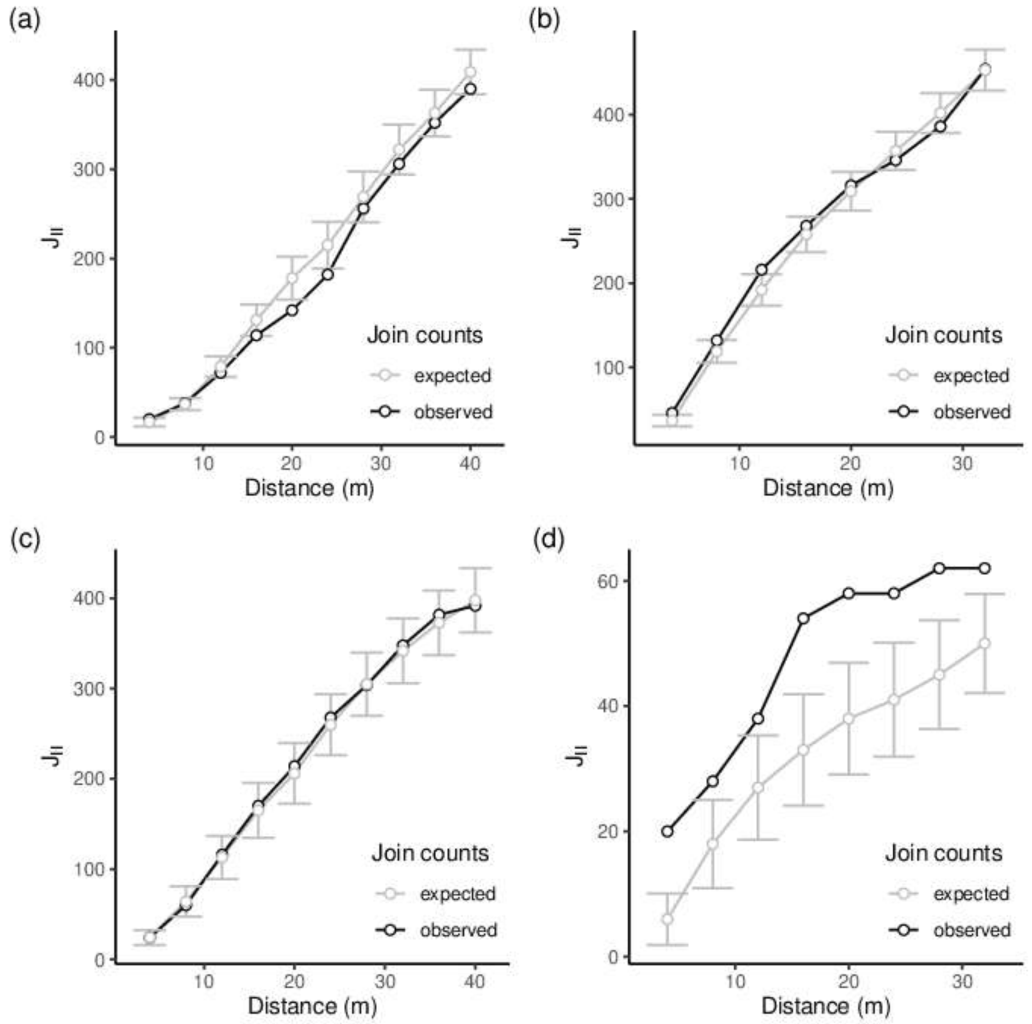

Supplement: Supplementary file 1 [file insects-14-00180-s001.zip › insects-2156765-supplementary/Figure_S1.pdf]
