# Supplementary material for: The Pattern of Social Parasitism in Maculinea teleius Butterfly Is Driven by the Size and Spatial Distribution of the Host Ant Nests
Source: Insects. 2023 Feb 12;14(2):180. doi: 10.3390/insects14020180 (PMC9961401; doi:10.3390/insects14020180)

Figure S2. Observed and expected values (with SD) of join count statistics (sum across nests) calculated for nests infested by *M. teleius*, relative to empty nests. The plots show the J_{II} values calculated in autumn (a,b) and in late spring (c, d). Plots on the left refer to Kraków (a, c,), while those on the right to Kosyń (b, d).

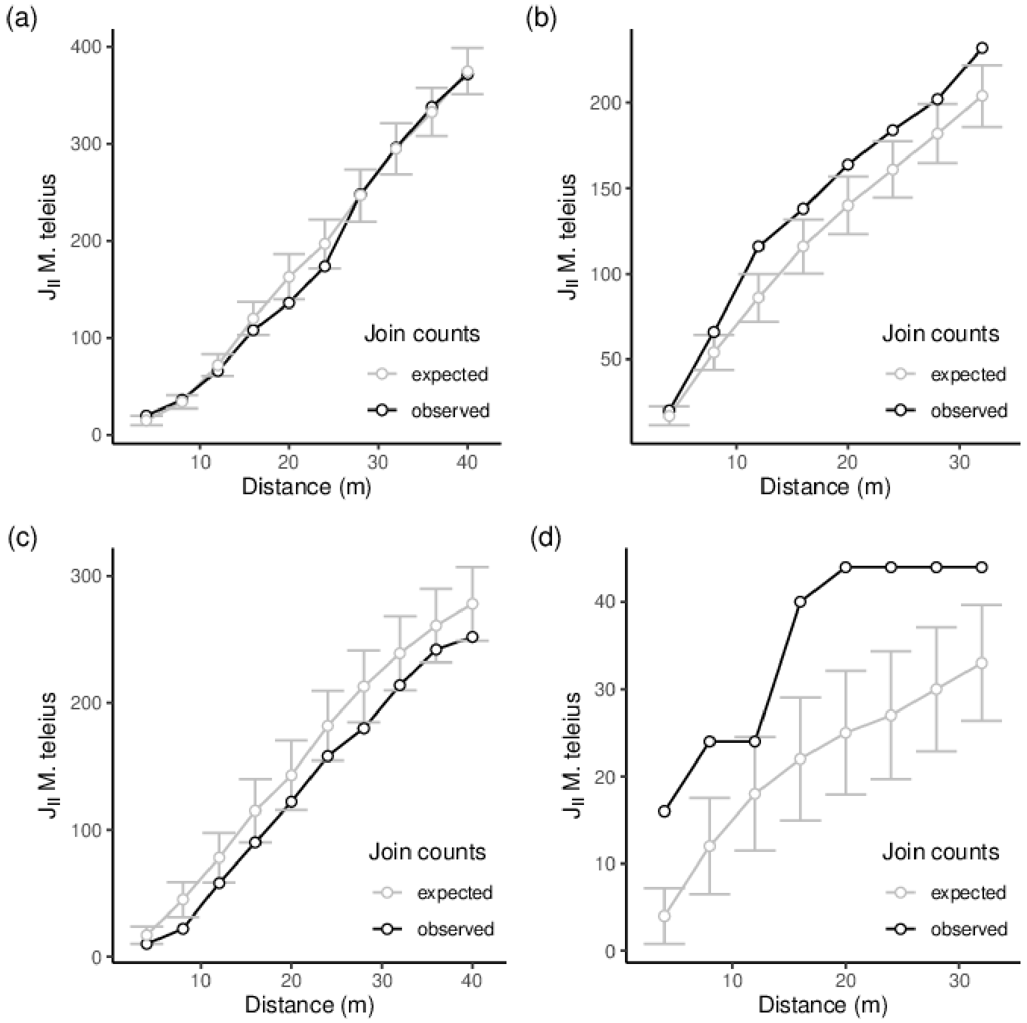

Supplement: Supplementary file 1 [file insects-14-00180-s001.zip › insects-2156765-supplementary/Figure_S2.pdf]
