# Supplementary material for: The Pattern of Social Parasitism in Maculinea teleius Butterfly Is Driven by the Size and Spatial Distribution of the Host Ant Nests
Source: Insects. 2023 Feb 12;14(2):180. doi: 10.3390/insects14020180 (PMC9961401; doi:10.3390/insects14020180)

Figure S3. Observed and expected values (sum) of join count statistics calculated for nests infested by *M. teleius* and/or other parasites for data collected in autumn at a) Kraków and b) Kosyń, and for the spring data set (c) at Kraków and (d) Kosyń. Values are plotted at several spatial distances and standard deviations are reported for the expected values. The J_{MtMt} statistics assess the occurrence of a positive association between *M. teleius* larvae, while the J_{MtO} statistics assess the likelihood of spatial segregation between *M. teleius* larvae and other parasite species (e.g., for positive spatial association, observed values must be significantly larger than expected).

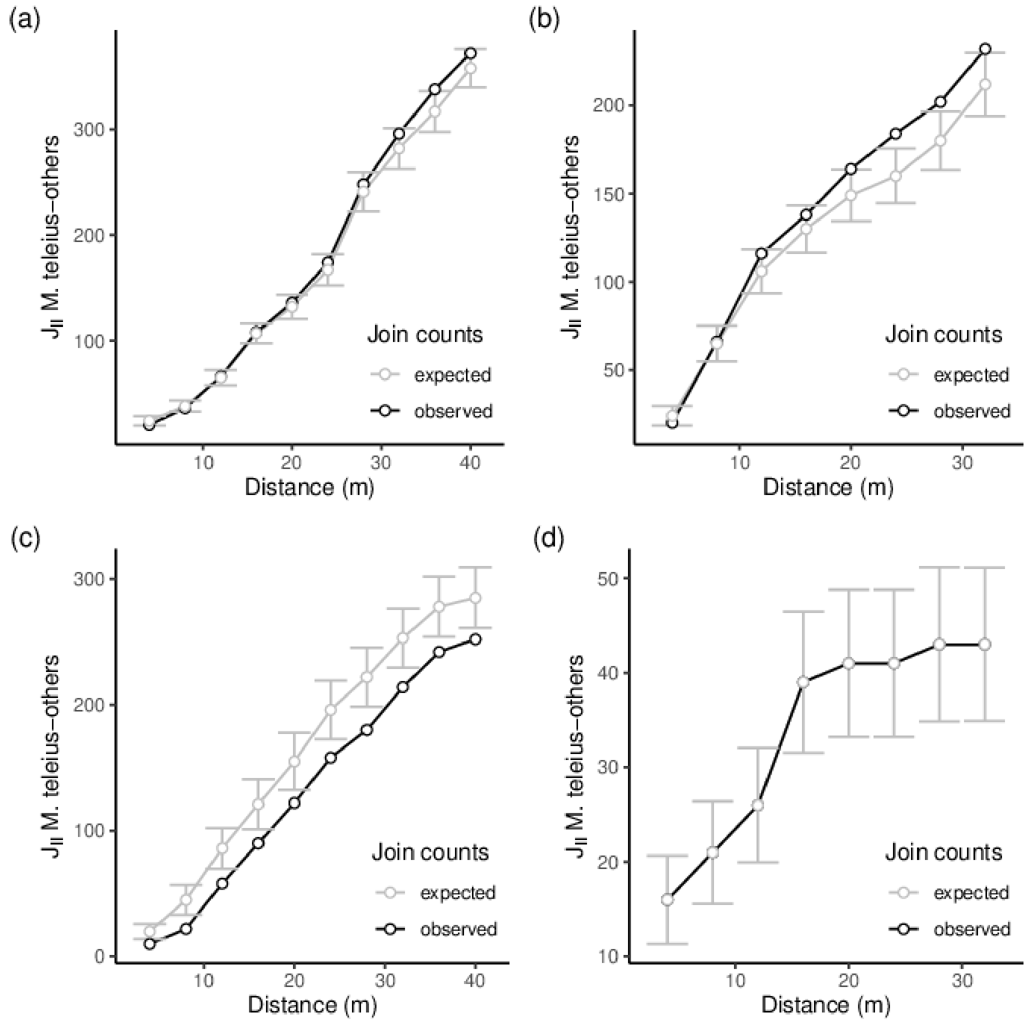

Supplement: Supplementary file 1 [file insects-14-00180-s001.zip › insects-2156765-supplementary/Figure_S3.pdf]
